# Ileocecal Lipoma as a Lead Point for Adult Ileocolic Intussusception: A Diagnostic Challenge

**DOI:** 10.1155/cris/4877273

**Published:** 2026-09-30

**Authors:** Sagar Kumar Jha, Shankar Kafle, Amit Kumar Thakur

**Affiliations:** ^1^ Tribhuvan University Teaching Hospital Maharajgunj Medical Campus Kathmandu Nepal teachinghospital.org.np; ^2^ Department of Pathology Tribhuvan University Teaching Hospital Maharajgunj Medical Campus Kathmandu Nepal teachinghospital.org.np; ^3^ Department of Pathology Madhesh Institute of Health Sciences Janakpurdham Madhesh Province Nepal

**Keywords:** acute abdomen, adult intussusception, giant colonic lipoma, hemicolectomy, ileocecal junction

## Abstract

Adult ileocolic intussusception caused by a giant ileocecal lipoma is an exceptionally rare cause of acute abdomen. We present the case of a 72‐year‐old male with ileocolic intussusception on CT scan who underwent manual reduction followed by definitive segmental resection with 2 cm margins. Histopathological examination revealed a large submucosal lipoma at the ileocecal junction acting as the lead point. This case highlights the importance of considering benign mesenchymal tumors in the differential diagnosis of acute abdomen in adults.

**Key Clinical Message**

• Intussusception is rare in adults and may present with an acute abdomen and mild anemia.
• A 72‐year‐old male presented with a large, globular, soft mass measuring 7.0 cm × 3.5 cm × 3.0 cm that was identified arising from the ileocecal junction, causing ileocolic intussusception.
• Preoperative differentiation between a benign lipoma and an underlying malignant neoplasm may be challenging. Histopathology remains the definitive tool to rule out malignancy and confirm the lipomatous nature of the lead point.

## 1. Introduction

Adult intussusception accounts for ~1%–5% of bowel obstructions, a stark contrast to the pediatric population, where the condition is usually idiopathic [1]. In adults, a demonstrable pathological lead point is identified in over 90% of cases, with nearly half of colonic cases being malignant [2].

Gastrointestinal lipomas are slow‐growing, benign adipose tumors. While often asymptomatic, lipomas exceeding 2 cm can cause symptoms such as pain, hemorrhage, or, rarely, serve as the lead point for intussusception [3]. The ileocecal region is a particularly rare site for such a large symptomatic lipoma, making this case a noteworthy contribution to the surgical literature [4].

## 2. Case Presentation

A 72‐year‐old male was admitted to the hospital with “abdominal pain for 5 days.” According to the patient, he had mild abdominal pain after eating for about 1 month; the pain was paroxysmal, dull pain with short duration and relieved spontaneously after about 5 min; he had no fever and chills, no nausea and vomiting, no obvious radiating pain, and no cessation of anal venting and defecation; the patient did not seek medical attention initially. Five days before admission, he was initially treated with analgesics at local medical shops, but the symptoms were not relieved; hence, he came to our hospital for treatment. He had no comorbidities or a history of previous surgery. On physical examination, his pulse rate was at the upper limit (96 times/min), while the temperature and respiratory rate were within normal limits. Abdominal examination revealed a bulge with tenderness in the left lower abdomen. There was no rebound tenderness or guarding, and shifting dullness was negative. Bowel sounds were hypoactive (3–4/min). There were no other findings, such as abdominal distension, vomiting, weight loss, or gastrointestinal bleeding.

On laboratory and imaging investigations, initial laboratory results revealed significant anemia and an elevated inflammatory response:

• Hemoglobin: 10.6 g/dL (mild anemia).
• Total leukocyte count: 9000/mm^3^ with neutrophilia (88.2%).
• Albumin: 32.5 g/L (mild hypoalbuminemia).
• CRP: 2.8 mg/dL (normal value is <1.0 mg/dL, thus indicating mildly raised, supporting low‐level inflammation).
• Procalcitonin: 0.08 ng/mL (normal).

A contrast‐enhanced CT scan of the abdomen revealed a fat‐density lesion in the small intestine in the left lower abdomen, suggestive of a lipoma acting as a lead point for ileocolic intussusception.

The patient underwent manual reduction, followed by exploratory laparotomy with right‐limited hemicolectomy and ileocolostomy. On pathological examination, the gross description includes the resected specimen, which consisted of parts of the cecum, terminal ileum, and ascending colon. A large, globular, soft mass measuring 7.0 cm × 3.5 cm × 3.0 cm was identified at the ileocecal junction. The external surface of the mass was smooth and shiny, with focal dull areas. Upon sectioning, the mass exhibited well‐circumscribed, yellowish‐white fatty tissue, as shown in Figure 1. The mass was situated 8.5 cm from the proximal resection margin and 7.5 cm from the distal resection margin. On histopathological examination, the mass was composed of a circumscribed proliferation of mature adipocytes separated by delicate fibrovascular septa (Figure 2). Histologically, no features of ischemia, gangrenous change, cellular dysplasia, or malignancy were observed. The epithelial lining of the colon and ileum was normal and columnar, and the subepithelium showed a moderate amount of mixed inflammatory cells. The postoperative outcome was good. He passed flatus and commenced a liquid diet on postoperative day 2. By day 3, he had a bowel movement and progressed to a semiliquid diet. He was discharged on the fifth postoperative day after tolerating a regular diet. Follow‐up at 2 and 12 weeks confirmed that the patient remained asymptomatic with no complications. Additional follow‐up was done after the 1‐year postoperative period via telephone. He remained asymptomatic, resumed usual daily activities with no signs and symptoms related to intussusception or bowel abnormalities, and overall had satisfactory functional recovery.

**Figure 1:**
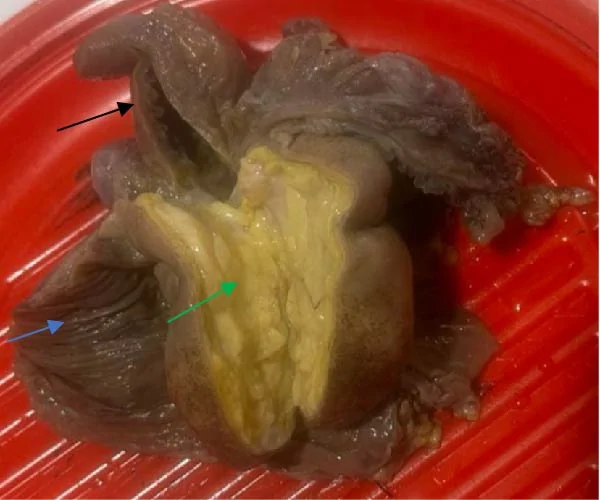
Gross image showing a large, globular, soft mass, with a yellowish and soft cut surface (shown by the green arrow) in the submucosa (black arrow: ileal part and blue arrow: colonic part).

**Figure 2:**
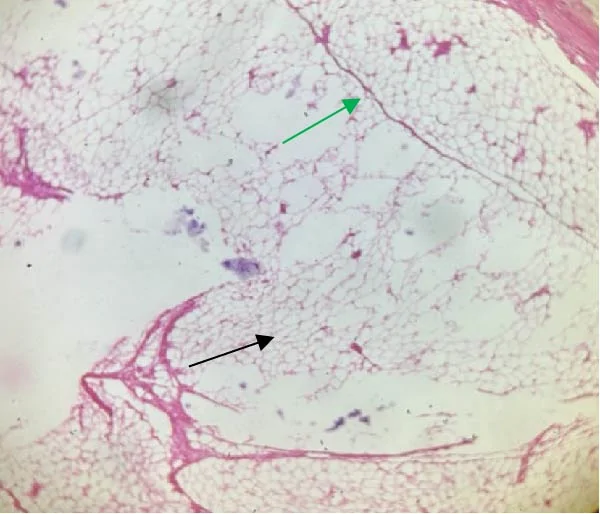
Microscopic view showing mature adipocytes (black arrow) arranged in lobules separated by delicate fibrovascular septa (green arrow), consistent with lipoma (H&E stain, low power).

### 2.1. Diagnosis and Challenges

The definitive diagnosis was intussusception triggered by a submucosal lipoma. The challenge in such cases is differentiating benign lipomas from malignant lesions (such as liposarcomas or adenocarcinomas), as imaging can be inconclusive and may require radical surgery rather than simple excision [5]. Histopathology is necessary for a definite diagnosis.

## 3. Discussion

Adult intussusception is a diagnostic chameleon, often presenting with nonspecific chronic abdominal pain before progressing to acute obstruction [6]. Unlike pediatric cases, where reduction is the standard, symptomatic adult cases generally require surgical resection due to the high incidence of lead‐point pathology, particularly appropriate when colonic involvement or malignancy is suspected [7]. Submucosal lipomas are the second most common benign tumor of the colon after adenomas [8]. When these masses reach a significant size, as in our patient (7 cm), gut peristalsis attempts to move the mass distally, dragging the attached intestinal wall behind it and resulting in ileocolic intussusception [9].

A CT scan can be a reliable indicator of intussusception, and radiographic images of a “target sign,” “bull’s eye,” or “doughnut sign” are auxiliary in diagnosing intussusception. However, imaging may not clarify the underlying etiology [10]. In our case, due to a lack of a proper data storage system in our institution, the original CT scan images could not be retrieved. However, the available CT scan report did not mention any additional information, such as target or Sausage sign, mesenteric vessel inclusion, or mesenteric fat and vessels within the intussusception. It was mentioned that there was no bowel obstruction. It also mentioned“lipoma as the lead point,” but CT attenuation values to support this could not be traced. We have taken the unavailability of radiological images as a study limitation.

In this case, the involvement of the terminal ileum and ascending colon warranted a right hemicolectomy, which allowed complete excision of the ileocecal lipoma and definitive treatment of the ileocolic intussusception despite the absence of bowel obstruction [11].

The rarity of this case lies in the transition of a typically “silent” benign tumor into a surgical emergency. Gastrointestinal lipomas are found in ~0.2%–4.4% of autopsies, yet the vast majority remain asymptomatic throughout a patient’s life [12]. A typical colonic lipoma usually presents as an incidental finding during colonoscopy; however, when the diameter exceeds 2 cm, the risk of signs and symptoms increases exponentially, such as disruption of normal intestinal motility, converting a stable submucosal mass into a mobile lead point. Peristaltic activity then attempts to propel this mass distally, ultimately precipitating intussusception [13, 14].

A notable clinical feature in our case was the presence of mild anemia (hemoglobin 10.6 g/dL). A case report by Li [15] mentioned that anemia in intussusception was associated with gastrointestinal bleeding. Further comparison of our case with previously reported literature based on age, sex, tumor size, anatomical location, surgical procedure, and postoperative outcome is shown in Table 1 [16–18].

**Table 1:** Representative reported cases of adult ileocolic intussusception secondary to giant lipoma.

Study	Age/sex (M = male and F = female)	Size (cm)	Anatomical Location	Surgical procedure	Outcome
Balamoun and Doughan [16]	49/M	5.0	Terminal ileum	Right hemicolectomy	Uneventful
Minaya Bravo et al. [17]	75/M	6.0	Ileum (ileocolic)	Right hemicolectomy	Recovered
Lee and Choe [18]	29/F	5.5	Terminal ileum	Ileocecal resection	Uneventful
Present case	72/M	7.0	Ileocecal junction	Right hemicolectomy	Well at 1 year

Immunohistochemistry was not performed in the present case because the histomorphological features were characteristic of a conventional lipoma and did not raise suspicion for an atypical lipomatous tumor/well‐differentiated liposarcoma (WDLPS) or another mesenchymal neoplasm. However, immunohistochemistry is very helpful, along with histological features, in cases of suspicion of mesenchymal tumors like gastrointestinal stromal tumor (GIST), WDLPS, as well as other malignancies like adenocarcinoma or lymphoma (Table 2) [19–22].

**Table 2:** Histological and immunohistochemical features of differentials of lipoma.

Differential	Histopathological distinction	Useful IHC if required
Lipoma	Mature adipocytes, no atypia/lipoblasts	Usually unnecessary
WDLPS	Atypical stromal cells/septa ± lipoblasts	MDM2, CDK4
GIST	Spindle/epithelioid cells	KIT, DOG1
Adenocarcinoma	Malignant glandular epithelial proliferation	Cytokeratins ± site‐specific markers
Lymphoma	Atypical lymphoid proliferation	CD45 + lineage markers

The surgical management of adult intussusception remains controversial, particularly regarding the decision between reduction and primary resection. Unlike pediatric intussusception, which is often idiopathic and managed conservatively, adult intussusception has an identifiable pathological lead point in 70% to 90% of cases, with up to 50% of colonic cases being malignant [1]. Consequently, most authors advocate for primary resection without attempted reduction.

In the present case, a cautious manual reduction, followed by definitive segmental resection with 2 cm margins, was performed. This strategy allowed preservation of bowel length while ensuring complete oncological clearance of the 7 cm lesion, thereby minimizing the risk of recurrence.

## 4. Conclusion

Lipoma‐induced intussusception in adults is a rare but critical differential diagnosis for an acute abdomen. Although lipomas are benign, their ability to cause significant mechanical complications often warrants surgical intervention. Histopathology remains the definitive tool to rule out malignancy and confirm the lipomatous nature of the lead point.

## Author Contributions

Sagar Kumar Jha contributed to the preparation of the original draft and review. Shankar Kafle contributed to the preparation of the original draft, collection of images, history taking, and other details of the patient, and review. Amit Kumar Thakur contributed to the preparation of the original draft and a review of the case.

## Funding

There is no funding received for this study.

## Ethics Statement

This study has been exempted from ethical approval by our institution. However, the patient provided written informed consent for publication and the use of case details, including images.

## Conflicts of Interest

The authors declare no conflicts of interest.

## Data Availability

Data will be made available upon request by the authors.
